# A Facile Strategy to Prepare Shaped ZSM-5 Catalysts with Enhanced Para-Xylene Selectivity and Stability for Toluene Methylation: The Effect of In Situ Modification by Attapulgite

**DOI:** 10.3390/molecules24193462

**Published:** 2019-09-24

**Authors:** Yiren Wang, Yang Chang, Min Liu, Anfeng Zhang, Xinwen Guo

**Affiliations:** State Key Laboratory of Fine Chemicals, PSU-DUT Joint Center for Energy Research, School of Chemical Engineering, Dalian University of Technology, Dalian 116024, China; wangyr@mail.dlut.edu.cn (Y.W.); changyang@cnooc.com.cn (Y.C.); lium@dlut.edu.cn (M.L.); zhangaf@dlut.edu.cn (A.Z.)

**Keywords:** toluene methylation, zeolite extrudates, para-xylene selectivity, attapulgite, binder, stability

## Abstract

A general strategy for preparing shaped toluene methylation catalysts with enhanced para-selectivity and stability is developed by extruding ZSM-5 zeolite with attapulgite as a binder. The novel attapulgite/ZSM-5 extrudate exhibited significantly higher para-selectivity and stability in comparison to the conventional alumina-bound ZSM-5 extrudate. The catalyst samples have been characterized by in situ X-ray diffraction, scanning electron microscope (SEM), NH_3_ temperature programmed desorption (TPD), thermogravimetric analysis (TGA) as well as n-hexane/cyclohexane physical adsorption. The enhanced catalytic performance of attapulgite/ZSM-5 extrudate is correlated with the in-situ modification of acid sites in the catalyst by mobile alkaline species, which is introduced via extrusion with attapulgite. Moreover, a higher para-selectivity was obtained over attapulgite-bound modified ZSM-5 extrudate. Such facile and universal strategy of extruding ZSM-5 catalysts with attapulgite as binder could pave a way for preparation of shaped zeolite-base catalyst with enhanced catalytic performance.

## 1. Introduction

Para-xylene, a critical raw material for polyester manufacture, is the most lucrative petrochemical commodity among all three xylene isomers (ortho-xylene, meta-xylene and para-xylene) [1,2,3,4]. The high profit of para-xylene production has created incentives for researchers to work on different pathways to obtain para-xylene. Among all the routes for producing para-xylene, toluene methylation with methanol to para-xylene is promising to become an important process in the chemical industry, because toluene is produced beyond market demand [4,5] and methanol is expected to be extensively synthesized from coal and natural gas [4,6].

Medium-pore zeolite ZSM-5 is an attractive para-selective alkylation catalyst because its pore size is comparable to para-xylene dimension [1,7]. However, mixed xylenes with composition close to thermodynamic equilibrium distribution (ortho:meta:para xylene ratio of ~22:53:25 [8]) are produced over unmodified ZSM-5 zeolites, especially nano-sized ZSM-5 zeolites [9]. Due to the similar boiling points, shapes and polarities of xylene isomers, para-xylene is separated from other xylene isomers through a high-cost and energy-intensive process of distillation, adsorption and cryogenic crystallization [10,11]. Thus, improving para-selectivity in xylene product, as an effective way to reduce the cost of para-xylene production, is the first priority for catalyst design. Commonly used techniques include impregnation with inorganic agents like boron, phosphorous or magnesium compounds [12,13,14], surface silylation by depositing teraethyl orthosilicate [9,12], tuning the crystal sizes of ZSM-5 [3] and pre-coking of ZSM-5 zeolites [5].

In most cases, zeolites are obtained and used in powder form for research purposes. To implement zeolites in large-scale reactors, the shaping process of zeolites (i.e., dispersing zeolites into binders and shaping to the desired shape) is required to avoid high-pressure drop in catalyst bed [15]. However, the preparation of zeolite catalysts from powder to industrially relevant shapes, and the influence of shaping on the resulting catalysts were largely neglected in academic investigations. Many recent academic studies have been devoted to understanding the impact of shaping process in the vibrant area of zeolite catalysis [15,16,17]. The zeolite-binder interactions result in multiple effects on catalyst activity, stability and product selectivity, with the type of binder playing a pivotal role [16,18,19,20,21].

Attapulgite, a fibrous like morphology clay, belongs to hydrated magnesium aluminum silicate minerals. There are significant reserves of attapulgite in China, America and Spain [22,23]. In recent years, attapulgite has received much attention from the academic world due to its structural morphology, surface properties, low cost and eco-friendly nature [23,24]. Attapulgite is mainly used as sorbent in the removal of metal ions [24,25], catalyst support for various reactions [26], polymer additive for mechanical improvement [27] and synthesis additive for new materials [22,28]. The abundant hydroxyl groups on attapulgite’s surface makes it a good binder as well [29]. These hydroxyl groups are dehydrated and cross-linked between adjacent binder particles after calcination, thereby increasing mechanical strength of the shaped catalyst [18].

In this study, a facile strategy for one-step preparation of shaped toluene methylation catalyst with enhanced para-selectivity and stability is developed by extruding ZSM-5 with attapulgite as binder. The major objective of this work is to evaluate the performance of ZSM-5/attapulgite extrudate in toluene methylation reaction. Compared with conventional binder boehmite, attapulgite binder improved the para-xylene selectivity and the life span of ZSM-5 zeolite catalyst without a significant deterioration of the catalytic activity. The in-situ modification effect of attapulgite was elucidated based on systematic characterizations and catalytic performance of the zeolite extrudates. The applicability of this strategy is also tested.

## 2. Results and Discussion

### 2.1. Preparation and Characteristics of ZSM-5 Extrudates

**Figure 1:**
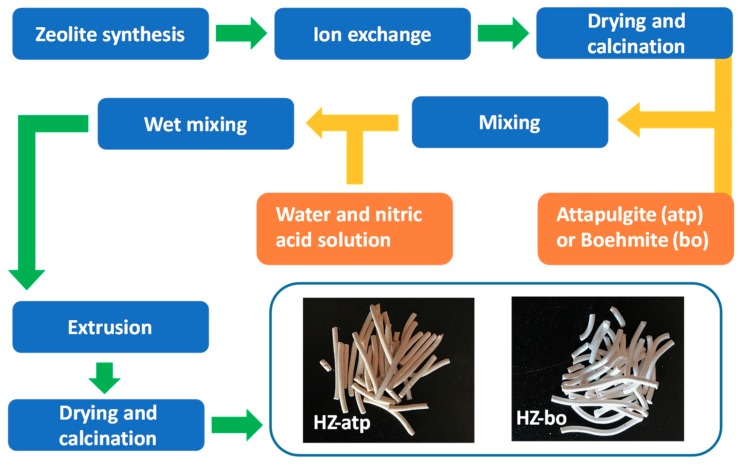
Steps in the preparation of zeolite extrudates with attapulgite and boehmite and key sample photos.

Two zeolite extrudates (the novel extrudate HZ-atp and the conventional extrudate HZ-bo) were obtained following a standard industrial protocol (Figure 1). The dry mass ratio of H-ZSM-5 zeolite and attapulgite or boehmite binders is 1:1. Herein, the compositional difference between two binders is noteworthy. Boehmite (AlOOH), a traditional binder, becomes aluminum oxide (Al_2_O_3_) after calcination in air [17]. Attapulgite, a natural clay, is composed of 66.4 SiO_2_/15.7 MgO/10.2 Al_2_O_3_/5.3 Fe_2_O_3_/1.5 CaO/0.9 K_2_O, revealed by X-ray fluorescence spectroscopy analysis.

**Figure 2:**
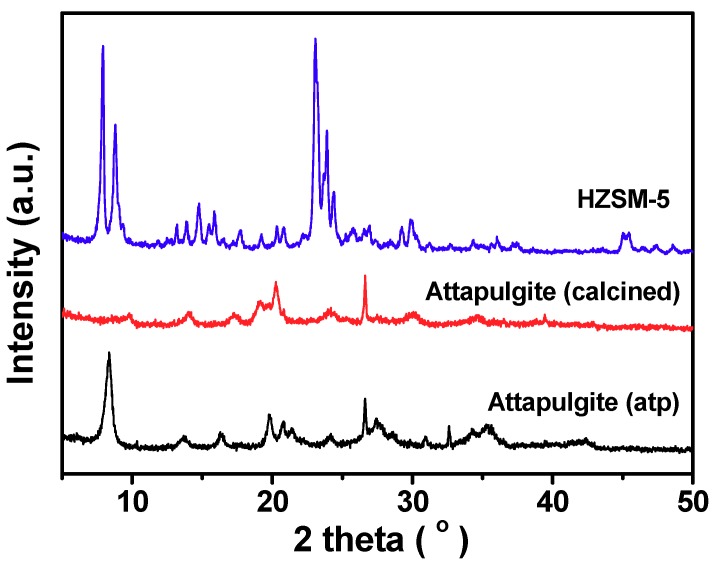
X-ray powder diffraction (XRD) patterns of H-ZSM-5 powder; the attapulgite binder before and after calcination

**Figure 3:**
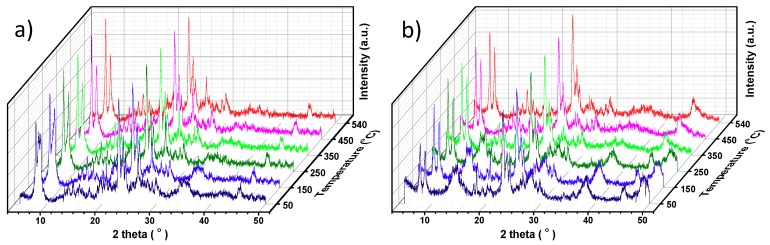
In situ XRD patterns of HZ-atp (**a**) and HZ-bo (**b**); in situ scanning at 100 °C steps from 50 °C to 540 °C under synthetic air.

X-ray powder diffraction (XRD) patterns of the H-ZSM-5 zeolite and the attapulgite binder are shown in Figure 2. The attapulgite (atp) is identified by its characteristic diffraction peaks at 8.4°, 13.8°, 19.8°, 27.3°, 35.4° and 42.6° (JCPDS: 21-0958). The calcination process causes the disappearance of attapulgite diffraction peaks and the growth of quartz phase (peaks at 20.3° and 26.6°, JCPDS: 46-1045), which indicates the crystalline structure of attapulgite was destroyed. The in situ XRD patterns of the dried HZ-atp and HZ-bo were collected during a stepwise temperature-programmed process (Figure 3). Evidenced by the fact that the five characteristic diffraction peaks of MFI topology at 7.8°, 8.8°, 23°, 23.9° and 24.4° (JCPDS: 44-0003) were discovered in every pattern in Figure 3, it is clear that the ZSM-5 structure was not damaged during extrusion or calcination process. However, the binders were gradually decomposed as the temperature raised. The crystalline structures of attapulgite and boehmite were greatly damaged after 350 °C. The in situ XRD patterns collected at 540 °C showed that the HZ-atp and the HZ-bo extrudates became a mixture of H-ZSM-5 zeolite and amorphous oxides after calcination.

**Figure 4:**
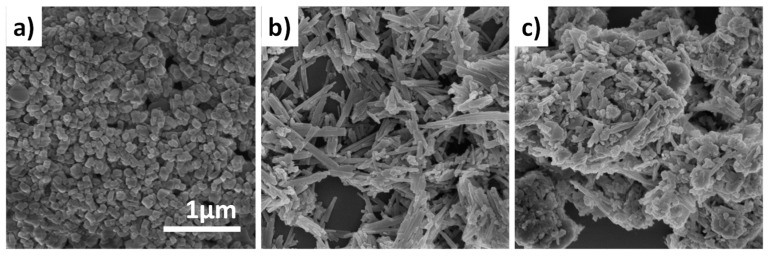
Scanning electron microscopy (SEM) images of H-ZSM-5 (**a**), attapulgite (**b**) and calcined HZ-atp extrudate (**c**). The scale bar represents 1 μm and applies to all images.

Figure 4 shows scanning electron microscopy (SEM) images of the H-ZSM-5 zeolite, the attapulgite binder and the calcined HZ-atp extrudate. The H-ZSM-5 zeolite is an aggregate of cubic shaped particles with crystal size of 100 nm. Attapulgite adopts a rod-like morphology with diameters of 40–60 nm and lengths of 200 nm to 2 μm. The morphology of calcined HZ-atp extrudate is a composite of H-ZSM-5 and attapulgite, in which the rod-like attapulgite binder particles wrap around H-ZSM-5 aggregates. An interesting observation is that the rod-like morphology of attapulgite remained after calcination even though its crystalline structure was destroyed. As a hydrous magnesium-aluminum silicate mineral (Al_2_Mg_2_Si_8_O_20_(OH)_2_(OH_2_)_4_·4H_2_O), the calcination process of attapulgite is mainly dehydration and dehydroxylation. Thus, the rod-like morphology could be preserved during calcination. The SEM image of HZ-atp also shows the intimate contact between the H-ZSM-5 particles and the highly dispersed attapulgite binder, suggesting the possible existence of zeolite-binder interactions.

**Figure 5:**
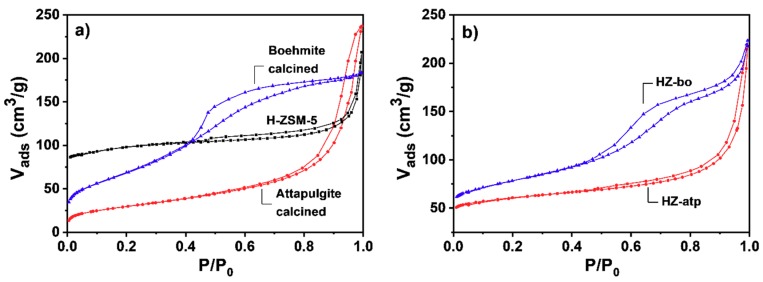
Nitrogen adsorption/desorption isotherms of powders (**a**) and extrudates (**b**).

To measure the textural properties of the pure H-ZSM-5 zeolite, the pure binders and the zeolite extrudates (HZ-atp and HZ-bo), N_2_ physical adsorption at −196 °C was employed. As shown in Figure 5, the N_2_ adsorption isotherm of the H-ZSM-5 zeolite powder exhibits type I(b) behavior, according to IUPAC classification [30]. The major uptake at low relative pressure (P/P_0_ < 0.01) is associated with micropore filling. The uptake at high relative pressure (P/P_0_ > 0.8) is referred to the intercrystalline mesopores and macropores caused by small crystal aggregation, which is observed in SEM. The N_2_ adsorption/desorption isotherms of the boehmite-derived Al_2_O_3_ binder powder belongs to type IV(a) isotherm. The first stage of adsorption uptake corresponds to the initial monolayer-multilayer adsorption on the mesopore wall [30]. The following uptake in the P/P_0_ range of 0.4–0.8 corresponds to capillary condensation, which is accompanied by a type H2(a) hysteresis loop. Such isotherms indicate that the boehmite-derived Al_2_O_3_ binder mainly contains mesopores. The N_2_ adsorption isotherm of the attapulgite-derived binder powder features type II characteristics. The gradually increasing adsorbed amount as a function of the relative pressure and vast uptake at high relative pressure indicates the presence of macropores in the material. The N_2_ isotherms of zeolite-binder composites HZ-atp and HZ-bo extrudates showed both characteristic features of the isotherms of H-ZSM-5 and the pure binders. Isotherm with shape that are intermediate between type I(b) and type II is observed on HZ-atp. Likewise, isotherm of HZ-bo features type I/IV characteristics. In both isotherms, the reduction in low relative pressure uptakes of HZ-atp and HZ-bo resulted from the incorporation of binders. The significant uptakes observed at high relative pressure are attributed to the filling of the mesopores and macropores introduced via binders. The BET (Brunauer, Emmett and Teller) surface areas of the extrudates (listed in Table 1) are slightly lower than the calculated surface areas based on the proportional contributions of the zeolite and binder phases. The micropore volume calculated using t-plot method was also listed in Table 1. The micropore volume of HZ-atp (0.07 cm^3^/g) was half of the micropore volume of H-ZSM-5 (0.13 cm^3^/g), while micropore volume of HZ-bo was lower (0.04 cm^3^/g). Such results imply that most micropores in H-ZSM-5 are accessible after the extrusion with attapulgite, since the extrudates contain 50 wt% H-ZSM-5 zeolite powder. However, some micropores in HZ-bo might be blocked after extrusion with boehmite.

**Figure 6:**
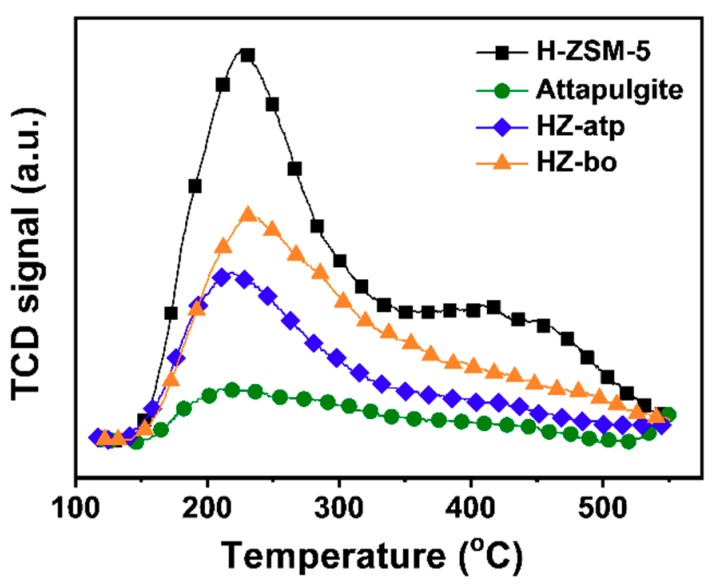
NH_3_-TPD profiles of H-ZSM-5 zeolite and HZ-atp and HZ-bo extrudates.

The acidity of the extrudate catalysts were investigated by using temperature-programmed desorption of ammonia (NH_3_-TPD). The information on the acid strength together with the amount of acid sites over solid acid catalysts can be derived from the NH_3_-TPD profiles. The NH_3_ desorption peak temperature reflects the acid strength and the area of desorption peak represents the quantity of acid sites. As shown in Figure 6, two major acid sites were identified on the H-ZSM-5 zeolite and the zeolite extrudates (HZ-atp and HZ-bo), i.e., weak acid sites (temperature peak at around 220 °C), and strong acid sites (temperature peak at around 430 °C). For ZSM-5 zeolite, the strong acid sites are mainly Brønsted acid [9]. The area of desorption peaks was integrated and calculated to determine the amount of total acid sites of the catalysts (presented in Table 1). The much less total acid sites of the zeolite extrudates HZ-atp and HZ-bo indicate the diluting effect of binders [31]. Compared with HZ-Bo, HZ-atp displayed a lower concentration of acid sites and weaker acid strength. Since no significant pore blocking was detected in the N_2_ physical adsorption of HZ-atp, it implies that part of the acid sites in HZ-atp were neutralized by some basic species in attapulgite (e.g., MgO) [21,29,32]. Interestingly, when the acid concentration was calculated based on mass of H-ZSM-5 zeolite in the extrudates, the acid sites increased after extrusion with boehmite. This indicates that new acid sites were introduced in the alumina-bound ZSM-5 extrudate.

### 2.2. Catalytic Performance in Toluene Methylation Process

**Figure 7:**
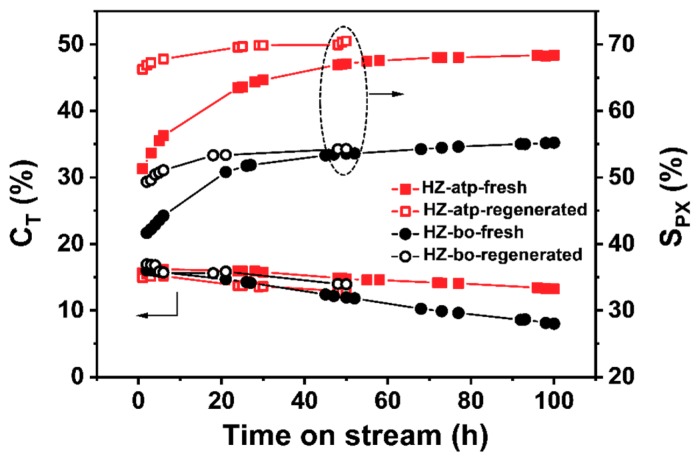
Toluene conversion and para-selectivity as a function of time on stream over HZ-atp (square) and HZ-bo (circle). Solid for fresh catalysts and open for regenerated catalysts.

The performance of the prepared zeolite extrudates and that of the pure powders were evaluated via the toluene methylation reaction (listed in Table 1). The results demonstrate that the binders (attapulgite and boehmite) did not exhibit catalytic activities for toluene methylation. To achieve an equivalent weight hourly space velocity with respect to the amount of zeolite used in the tests, the mass of the pure H-ZSM-5 was half of the mass of zeolite extrudates used in the reaction, since the extrudates contained 50 wt% of binders. The H-ZSM-5 zeolite catalyst exhibited a toluene conversion of 16.4%, but its para-selectivity was 30.0% (close to the para-xylene concentration in a thermodynamic equilibrium mixture of xylenes). In comparison, the zeolite extrudates exhibited much higher para-selectivity (i.e., 55.9% for HZ-atp and 43.5% for HZ-bo) with a slight reduction on toluene conversion (i.e., 16.1% for HZ-atp and 15.9% for HZ-bo). Figure 7 depicts the plot of toluene conversion and para-selectivity as a function of time on stream (TOS) over the two zeolite extrudates. Both HZ-atp and HZ-bo showed the trends that toluene conversion decreased with TOS and para-selectivity increased with TOS. For fresh zeolite extrudates, the initial toluene conversions were both 16.1% for HZ-atp and HZ-bo. The initial para-selectivities were 53.7% for HZ-atp and 42.2% for HZ-bo. After 100 h on stream, the para-selectivity reached 68.3% for Z5-atp and 55.2% for Z5-bo. Meanwhile, the toluene conversion decreased to 13.3% for Z5-atp and 8.0% for Z5-bo. The novel zeolite extrudate HZ-atp exhibited higher para-selectivity and more excellent stability than the conventional zeolite extrudate HZ-bo. The superior catalytic performance of HZ-atp further suggests the modification effect of attapulgite binder on the H-ZSM-5 zeolite, which is in accordance with the NH_3_-TPD results.

The commonly recognized catalyst design strategy for selective production of para-xylene in toluene methylation reaction is to deactivate the acidic sites on the external surface and reduce the catalyst pore openings. The deactivation of external acid sites hinders the undesirable isomerization of p-xylene product diffusing out from the pores. The reduced pore openings increase diffusional resistance of m- and o-xylene compared to p-xylene, making p-xylene the kinetically preferred product. Some researchers’ work has illustrated that Brønsted acid sites in zeolite extrudates could be partially neutralized due to the solid-state ion-exchange with mobile alkaline species in binders, such as sodium in montmorillonite [32] or magnesium in attapulgite [21,29]. As revealed in SEM images, the attapulgite binder in HZ-atp wrapped around the H-ZSM-5 zeolite particles, showing an intimate zeolite-binder interaction. Thus, it is reasonable to assume that the partial neutralization resulting from the zeolite-binder interaction started at the external surface of H-ZSM-5 zeolite. The enhanced para-selectivity of HZ-atp could be attributed to the partial neutralization of external acid sites.

**Figure 8:**
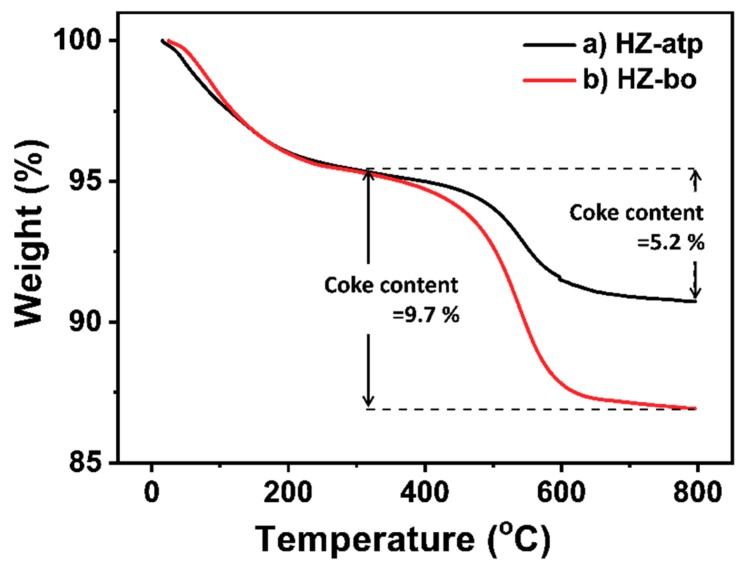
Thermogravimetric analysis (TGA) curves of HZ-atp (black) and HZ-bo (red) after toluene methylation reaction. Coke content was calculated by ([mass of 300 °C] – [mass of 800 °C])/[mass of 800 °C] × 100%.

After continuously operating for 100 h, spent catalysts were unloaded and their coke contents were investigated using thermogravimetric analysis (TGA) in an air flow. TGA curves of these spent catalysts are shown in Figure 8. Both spent catalyst samples displayed two distinct weight loss regions. The first weight loss occurred in the range of 25 to 300 °C, which was attributed to desorption of water and volatile species (i.e., reactants, products and reaction intermediates) adsorbed on catalyst surface [14,33]. The second weight loss was in the range of 300 to 800 °C, which was ascribed to the decomposition of coke species deposited on catalyst surface [14,33]. The coke content on spent HZ-atp was 5.2%, much less than that on HZ-bo. Prior studies have shown that extra-framework aluminum species in H-ZSM-5 catalyst favor the formation of coke by enhancing the oligomerization and hydrogen transfer reactions in methanol to hydrocarbons (MTH) process [16,29]. The HZ-atp extrudates contain less extra-framework aluminum species introduced in extrusion than the alumina-bound extrudates HZ-bo. Thus, the HZ-atp extrudates showed that less coke was generated during toluene methylation. Moreover, it is reported that mass transfer within shaped zeolite catalysts is dominated by diffusion in the macropores at elevated temperature [16,34]. In this regard, diffusivity of bulky molecules (e.g., coke precursor) in HZ-atp are more enhanced than HZ-bo because more macropores are introduced via extrusion with attapulgite, resulting in a slower coke formation rate.

### 2.3. In-situ Ion Exchange Modification Effect of Attapulgite

As shown in Figure 7, para-selectivity increased slowly and toluene conversion decreased slowly over both zeolite extrudates with time on stream (TOS). Such performance is commonly observed in toluene methylation reaction, which could be owing to the coke species generated during reaction deposited on catalyst surface and blocking some external acid sites and pore openings [5]. The TGA results showed that the coke content of HZ-bo was higher than HZ-atp (Figure 8). With regard to the coke content, the para-selectivity gap should become smaller after 100 h on stream if coking is the only cause for para-selectivity increment. However, it is observed that the initial para-selectivity gap between HZ-atp and HZ-bo was 11.5%, while this gap went up to 13.1% after 100 h on stream. Thus, there were other causes for the enhanced para-selectivity during reaction.

As mentioned in Section 2.2, earlier studies have found that solid-state ion exchange between protons of the zeolites and mobile alkaline species of the binder occurred during the calcination process subsequent to the extrusion, leading to the decrease of strong acid sites in zeolite catalysts [32]. Breen et al. have also shown that boron modified ZSM-5 can be produced in situ by either placing a physical mixture of boric acid and ZSM-5 in the reactor at the reaction temperature or placing a boric acid layer directly upstream of ZSM-5 bed [13]. Therefore, it is reasonable to assume that Mg species or other alkali oxides presented in attapulgite can also neutralize some of the acid sites in H-ZSM-5 zeolite throughout the reaction. In toluene methylation reaction conditions, steam is co-fed with reactants. The flushing down steam in reactor would be a help for ion migration.

**Figure 9:**
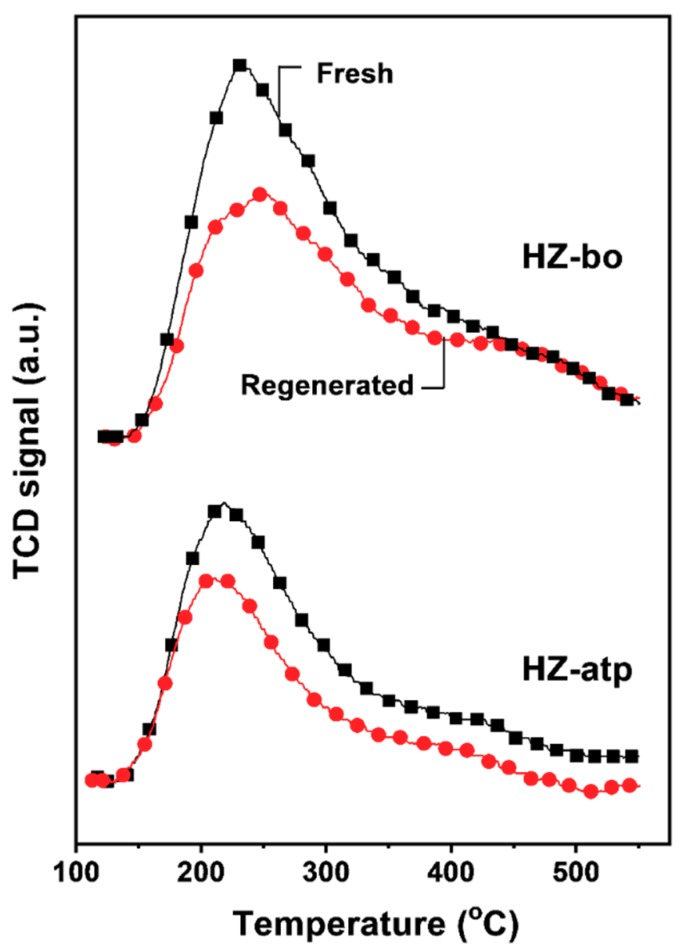
NH3-TPD profiles of fresh (black) and regenerated (red) zeolite extrudates.

To prove the in-situ modification hypothesis, the reusability of the two zeolite extrudates were tested. Spent HZ-atp and HZ-bo were regenerated at 540 °C under air atmosphere for 4 h. The catalytic performance of regenerated catalysts is shown in Figure 7. The toluene conversions of regenerated catalysts were equivalent to their fresh analogue. In the second cycle, the initial para-selectivity of regenerated HZ-bo was 49.3%, which was lower than the 55.2% para-selectivity after the first cycle, and the para-selectivity of regenerated Z5-bo after 50 h on stream was similar to its fresh analogue. In comparison, the initial para-selectivity of HZ-atp after regeneration was 66.2%, similar to its para-selectivity before regeneration. Moreover, the para-selectivity of regenerated HZ-atp continuously increased with TOS and reached 70.5% after 50 h, higher than the 68.3% para-selectivity after its first cycle. The different changes in para-selectivity over HZ-atp and HZ-bo before and after regeneration suggest an in-situ modification effect of attapulgite. To obtain more evidence of the in-situ modification assumption, acidity changes of fresh and regenerated zeolite extrudates were characterized by NH_3_-TPD. As shown in Figure 9, the amount of total acid sites of HZ-bo and HZ-atp both decreased after regeneration. The lower acidity of regenerated HZ-bo was mainly attributed to the decrease in weak acid sites, whereas both weak and strong acid sites decreased after the regeneration of HZ-atp. These changes in acidity are consistent with the para-selectivity variation observed in catalyst reusability test and the in-situ modification effect of attapulgite.

**Figure 10:**
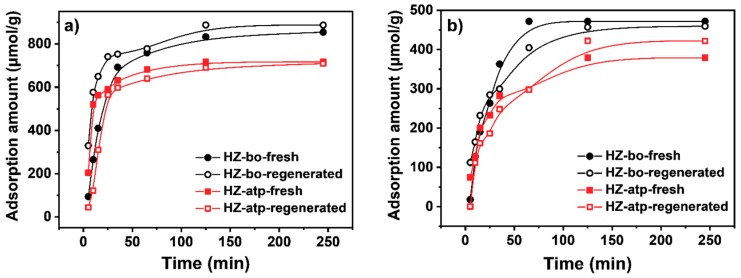
n-Hexane (**a**) and cyclohexane (**b**) adsorption of fresh and generated zeolite extrudates.

It is well known that in addition to the reduction of acidity, the zeolite modification with oxide may also reduce the effective dimensions of the catalyst pore openings [9]. The restricted pore openings increase diffusional resistance and, consequently, the para-selectivity in toluene methylation is improved. The extent of pore openings reduction of regenerated zeolite extrudates was evaluated by using n-hexane and cyclohexane adsorption experiments. n-Hexane can enter the channel of ZSM-5 zeolite (a ten-member ring channel) readily while the diffusion of cyclohexane in a ten-member ring channel is limited [35]. Therefore, the adsorption ratio of n-hexane to cyclohexane over samples is an indicator for the reduction extent of the pore openings [9,35]. The higher ratio means the larger reduction extent. Figure 10 shows the adsorption isotherms of n-hexane and cyclohexane over the fresh and regenerated zeolite extrudates. All extrudates exhibited similar adsorption rates, yet the saturation adsorption amounts of n-hexane or cyclohexane for four zeolite extrudates were different. Calculated from the saturation adsorption amounts, the n-hexane to cyclohexane adsorption ratio of HZ-bo increased from 1.8 to 1.9 after regeneration, whereas that of HZ-atp decreased from 1.9 to 1.7 after regeneration. It means that the initial para-selectivity increment for regenerated Z5-bo was not only due to the reduced acidity but partially due to the more restricted pore openings, which might be caused by the aluminum species migration in alumina-bound ZSM-5 extrudates [20,31,36]. In the HZ-atp case, the extent of pore openings reduction decreased after regeneration, which indicated that the in-situ modification of acidity was the main reason for the increase of para-selectivity over HZ-atp.

### 2.4. Catalytic Performance of Attapulgite-Bound Modified ZSM-5 Extrudates

**Figure 11:**
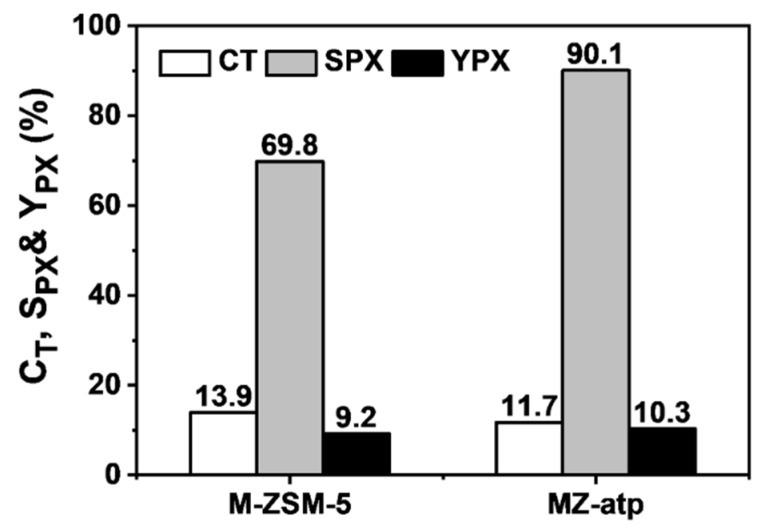
Toluene conversion, para-selectivity and yield of para-xylene over modified ZSM-5 (M-ZSM-5) and its extrudate with attapulgite (MZ-atp). Reaction conditions: 460 °C, atmospheric pressure, WHSV = 15 h^−1^, nT/nM = 4, nH_2_/n(T + M) = 2, nH_2_O/n(T + M) = 2.

In previous sections, it has been shown that extrusion with attapulgite is a facile strategy to obtain shaped catalysts with good para-selectivity. We expected that this strategy could be applied to other powder toluene methylation catalysts as well. In order to test its applicability, a Si-P-Mg modified ZSM-5 catalyst (M-ZSM-5) was extruded with attapulgite as binder. According to our previous investigations, the combined modification by Si, P and Mg with a proper sequence can efficiently reduce external surface acid sites and simultaneously narrow the catalyst pore openings, which lead to a higher para-selectivity [9,37,38]. Figure 11 shows the catalytic performance in toluene alkylation with methanol over M-ZSM-5 and the corresponding extrudate catalysts (MZ-atp). It is shown that the para-selectivity increased from 69.8% to 90.1% after extrusion. Such a result concludes that the modification effect of alkaline oxide species in attapulgite can apply to not only H-ZSM-5, but also a modified ZSM-5 catalyst. Though the para-selectivity of M-ZSM-5 extrudate increased at the expanse of toluene conversion, the yield of para-xylene increased significantly after extrusion with attapulgite.

## 3. Experimental

### 3.1. Catalyst Preparation

Na-ZSM-5 zeolite (SiO_2_/Al_2_O_3_ = 26) was synthesized according to the procedures reported previously [39]. Na-ZSM-5 was exchanged into its ammonium form by ion-exchange at 80 °C for 2 h with 1 M NH_4_NO_3_ solution (solid/liquid ratio, 10 g/50 mL). The solid was separated from the slurry by centrifugation and washed with deionized water thoroughly. The ion-exchange procedure was repeated four times. The product was then dried at 120 °C for 12 h and calcined at 540 °C for 4 h to remove all ammonia and produced the protonic form of ZSM-5 (H-ZSM-5). The binders investigated in this study included attapulgite (coded atp) and boehmite (coded bo). H-ZSM-5 and binder (1:1 dry mass ratio, accounting for the weight loss upon calcination to 540 °C) were blended thoroughly and then a predetermined amount of 0.5 M HNO_3_ solution and water was added into the mixture to form a paste for extrusion. The resulting extrudates were dried at 120 °C for 12 h and calcined in air at 540 °C for 4 h. The obtained catalysts using attapulgite and boehmite as binders were coded as HZ-atp and HZ-bo. For further investigations, H-ZSM-5 was modified with 6 wt% SiO_2_, 5 wt% P_2_O_5_ and 3 wt% MgO according to procedures described in our previous work [9,38], coded M-ZSM-5. The extrudate obtained by the above-mentioned procedure using M-ZSM-5 and attapulgite was coded MZ-atp.

### 3.2. Catalyst Characterization

Powder X-ray diffraction (XRD) was measured with a Rigaku SmartLab (9) diffractometer, using a nickel-filtered Cu Kα X-ray source at a scanning rate of 8°/min between 5° and 50°. In situ X-ray diffraction (IXD) was also carried out on the same equipment using an in situ reactor XRK-900. The temperature of the reactor was raised from 50 °C to 540 °C under synthetic air at a flow rate of 50 mL/min. During this process, the IXD patterns were collected every 100 °C. SEM images were taken using a field-emission scanning electron microscopy (NOVA NanoSEM 450) at an accelerating voltage of 10.0 kV. N_2_ adsorption/desorption isotherms at −196 °C were acquired with a Quantachrome Quantasorb-SI gas adsorption analyzer. The samples were degassed at 300 °C for 10 h before each run. Temperature programmed desorption of ammonia (NH_3_-TPD) was performed on an automated chemisorption analyzer (Quantachrome ChemBET Pulsar TPR/TPD) from 120 to 650 °C at a ramping rate of 10 °C/min. Thermogravimetric analysis (TGA) was carried out on a SDT Q600 thermal gravimetric analyzer (TA Instruments) in the temperature range of 25–800 °C under synthetic air atmosphere at a ramping rate of 10 °C/min. Isothermal adsorption of n-hexane and cyclohexane were measured on our homemade analyzing apparatus by using a flow gravimetric method at 25 °C. The sample was pretreated at 350 °C under nitrogen atmosphere for 1 h before measurement.

### 3.3. Catalytic Studies

The gas phase alkylation of toluene (T) with methanol (M) was studied in a typical down-flow fixed-bed reactor at 460 °C under atmospheric pressure. In each test, 0.5 g of the catalyst was employed and treated at 500 °C for 1 h under a hydrogen flow prior to reaction. The mixture of toluene and methanol (molar ratio 4:1) was introduced via an HPLC pump with a WHSV (weight hourly space velocity of toluene and methanol) of 15 h^−1^. Hydrogen and steam were used as carrier gas with molar ratios of H_2_/(T + M) = 2 and H_2_O/(T + M) = 2. The effluent from the reactor was collected in a cold trap and analyzed by gas chromatography (Agilent GC6890) equipped with a flame ionization detector (FID) and an INNOWAX capillary column (60 m × 0.25 mm × 0.25 µm).

The toluene conversion (C_T_), the para-selectivity (S_PX_) and the yield of para-xylene were defined in the following equations:

(1) $$C_{T}\;\left(\%\right)=\left(1-\frac{\mathrm{toluene}\;\mathrm{in}\;\mathrm{product}}{\mathrm{toluene}\;\mathrm{in}\;\mathrm{reactant}}\right)\times 100\%$$

(2) $$S_{\mathrm{PX}}\;\left(\%\right)=\frac{\mathrm{para}-\mathrm{xylene}}{\mathrm{para}-\mathrm{xylene}+\mathrm{meta}-\mathrm{xylene}+\mathrm{ortho}-\mathrm{xylene}}\times 100\%$$

(3) $$Y_{\mathrm{PX}}\;\left(\%\right)=\frac{\mathrm{para}-\mathrm{xylene}}{\mathrm{toluene}\;\mathrm{in}\;\mathrm{reactant}}\times 100\%$$

## 4. Conclusions

Binders are a vital component for the technical application of zeolite catalysts. The significant impact of the novel attapulgite binder on the shaped toluene methylation catalysts has been demonstrated. Compared with the traditional binder boehmite, alkaline oxides species present in attapulgite are able to modify the acid sites of zeolites during calcination process and throughout the reaction. The decreased acidity caused by the in-situ modification is correlated with the enhanced para-selectivity in toluene methylation reactions. Moreover, the macropores introduced via extrusion with attapulgite enhanced the mass transfer within shaped zeolite catalysts. As a consequence of the modified acidity and enhanced diffusivity, the novel attapulgite-bound extrudates of ZSM-5 showed a slow rate of coke formation and a prolonged life span. Other than attapulgite/H-ZSM-5 extrudate, a higher para-selectivity was obtained over attapulgite-bound modified ZSM-5 extrudate. The facile and universal strategy of extruding ZSM-5 catalysts with attapulgite as binder will open new routes for optimizing the performance in shaped catalysts.

## Figures and Tables

**Table 1:** Textural properties, acid sites concentration, and catalytic performance of the pure powder and extruded zeolite catalysts.

Catalyst	S_BET_^a^	V_total_^b^	V_micro_^c^	C_acid_^d^	C_acid_^d^	C_T_^e^	S_PX_^e^	Activity Loss ^f^
	(m^2^/g)	(cm^3^/g)	(cm^3^/g)	(μmol/g)	[μmol/g(ZSM-5)]	(%)	(%)	(%)
H-ZSM-5	376	0.21	0.13	385	385	16.4	30.0	--
Attapulgite	107	0.23	0	--	--	0.2	58.2	--
Boehmite	255	0.28	0	--	--	0.2	54.5	--
HZ-atp	227	0.22	0.07	153	306	16.1	55.9	17.5
HZ-bo	283	0.28	0.04	235	470	15.9	43.5	50.1

^a^ BET surface area calculated from the adsorption branch; ^b^ pore volume estimated from the single-point amount adsorbed at P/P_0_ = 0.95; ^c^ micropores volume calculated using t-plot method; ^d^ concentration of total acid sites derived from the NH_3_-TPD; ^e^ toluene conversion and para-selectivity are the average of samples collected from 4–6 h on stream; ^f^ activity loss: [(toluene conversion _initial_- toluene conversion _100 h_)/toluene conversion _initial_] × 100%.
